# Supplementary material for: Insight into the Lubrication and Adhesion Properties of Hyaluronan for Ocular Drug Delivery
Source: Biomolecules. 2021 Sep 30;11(10):1431. doi: 10.3390/biom11101431 (PMC8533502; doi:10.3390/biom11101431)
Supplement: Supplementary file 1 [file biomolecules-11-01431-s001.zip › biomolecules-1349010-supplementary.pdf]

Insight into the lubrication and adhesion properties of hyaluronan for ocular drug delivery

Mikuláš Černohlávek[1,2], Martina Brandejsová[1], Petr Štěpán[1], Hana Vagnerová[1], Jaromír Kulhánek[1], David Nečas[2], Martin Vrbka[2], Gloria Huerta Angeles[1*]

[1]Contipro a.s, Dolní Dobrouč 401, 561 02 Dolní Dobrouč, Czech Republic

[2]Faculty of Mechanical Engineering, Brno University of Technology, Technická 2896/2, 616 69 Brno, Czech Republic

[*]Correspondence: huerta-angeles@contipro.com

Tel.: + 420-465-519-573

S1 Transmittance of the solutions

The optical transmittance of the solutions based on derivatives of the examples above presented was analyzed with a UV-Vis spectrometer (**Fig. S1**). The percentage transmittance of the formulation in the range 200–800 nm, registered with respect to distilled water is also reported.

The turbidity of the samples was determined by preparing a solution (0.25 % wt) and adding into a quartz cell. The absorbance was measured at 860 nm with a Turbidimeter (TB 300IR) with light source IR-LED (860 nm) used in the range of 0.01 - 1100 NTU with an accuracy of ± 2% of reading (0-500 NTU)visible spectrophotometer as a function of time at 24 °C. The turbidity was calculated from standard absorbance–turbidity curve with formazin suspension as standards. Formazin suspension was prepared by reacting hydrazine sulfate with hexamethylenetetrammonium, and standards of formazin turbidity units (NTU) was prepared by appropriate dilution.

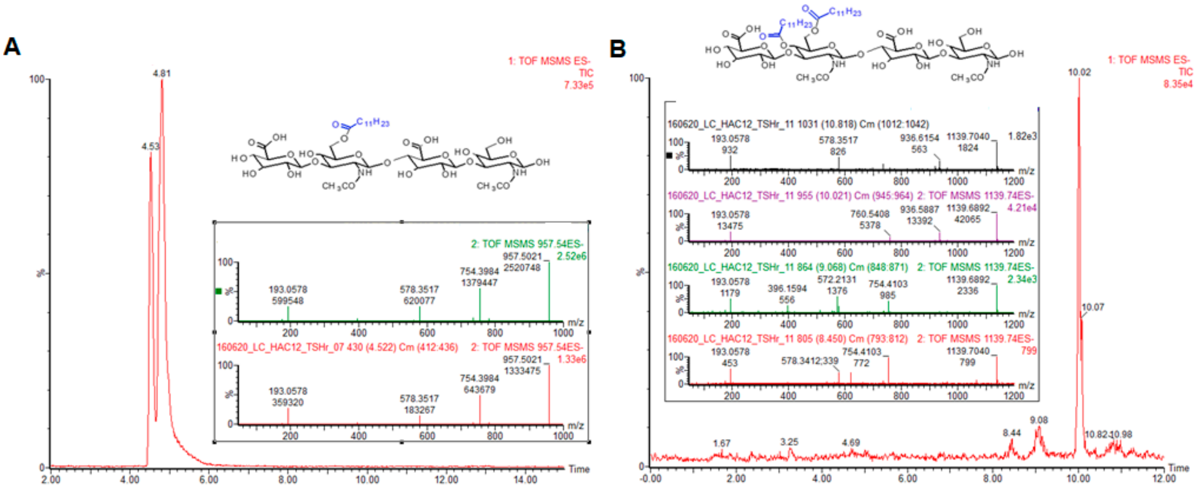

Figure S1. Normalized weight abundance of the mass contribution of the fragments: ΔHA4-C12 and ΔHA4-*2C12 obtained after enzymatic degradation.

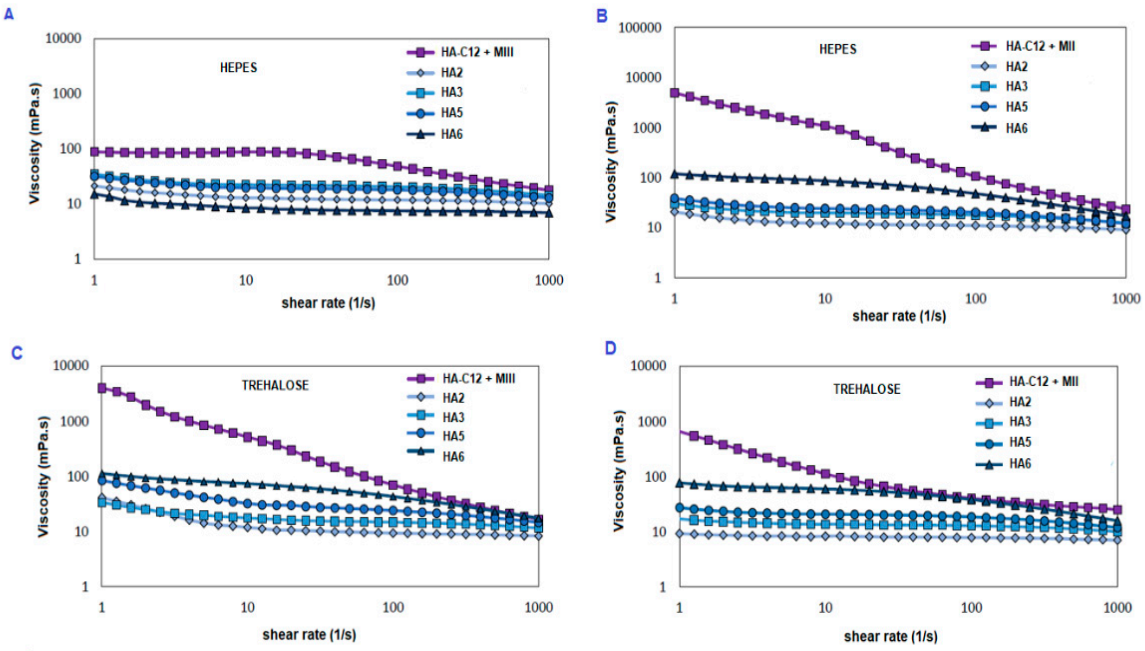

Figure S2. Rheological synergism for HA2-C12 and HA (tested with mucin II (B,D) or III (A,C) in HEPES or trehalose buffers at 37°C. All the samples used a constant concentration 0.3 wt. %.

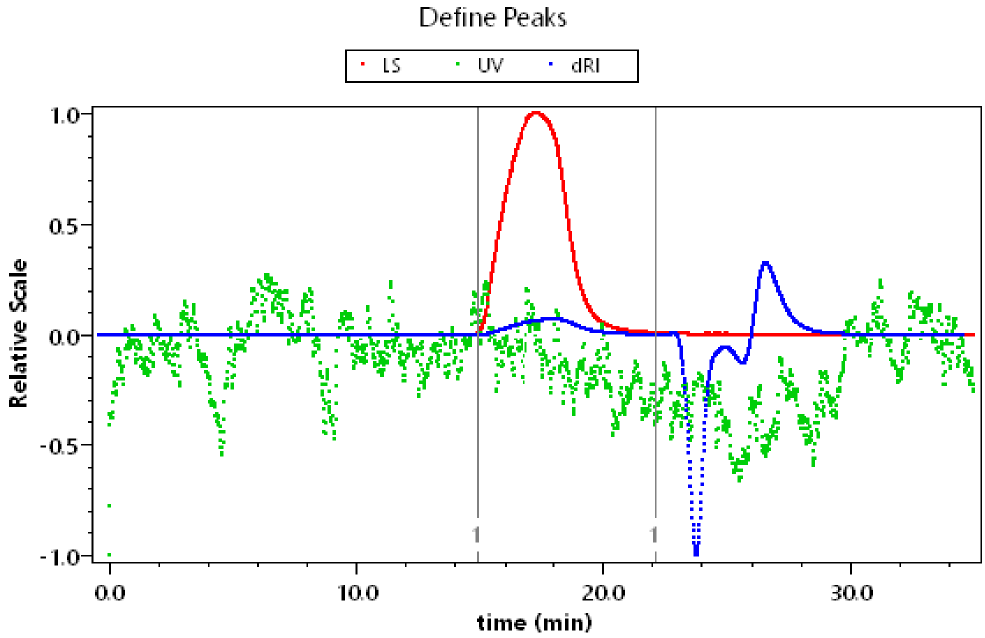

Figure S3. Determination of the MW for HA presented in Laimcare®.

S2. Turbidimetric titration

The interactions between mucin and HA and its derivatives were investigated by turbidimetric titration. Polymer solutions were added to 2 mL of the mucin dispersion stepwise and the turbidity (apparent absorbance) of the samples was measured after each step in an Agilent Cary 60 (Agilent) UV-Vis spectrophotometer at 500 nm. The absorbance values were recorded after reaching equilibrium.

Mucoadhesive interactions of HA and HA-C12 was studied by turbidity. 45 mg of HA was solubilized in 7.5 mL of HEPES buffer for 20 h at room temperature. Consequently 150 mg of mucin was dispersed in 7.5 mL of the same buffer. After that, the solutions were mixed (1:1 *v*/*v*) to obtain a final concentration of 1% of mucin and 0.3 wt. % of HA. The samples were incubated at 35 °C for 60 min. The absorbance and transmittance of the samples were measured at 500 and 660 nm, respectively (**see Table 2, S1**). To understand the effect of the chemical modification: **HA-C12** was measured at different concentrations 0.15, 0.3 or 0.5 wt. % as found in eye drops.

Table S1. Turbidimetric titration for native HA in HEPES buffer

Sample	Conc. (%)	Mucin	Turbidity	Absorb. 500 nm	Transmit. 500 nm	Absorb. 660 nm	Transmit. 660 nm
**HEPES buffer**		---	0.34	0.001	99.8	0.000	99.8
**Mucin III**	1	---	601.7	1.730	1.8	1.314	4.8
**HA2**	0.3	---	0.89	0.006	98.6	0.003	99.2
**HA3**		---	0.86	0.005	99.0	0.003	99.4
**HA4**		---	0.65	0.002	99.6	0.001	99.7
**HA5**		---	0.96	0.004	99.0	0.002	99.5
**HA6**		---	0.71	0.006	98.8	0.004	99.2
**HA2**	0.3	Type III	538.67	1.714	2.0	1.273	5.2
**HA3**			594.33	1.755	1.8	1.317	4.7
**HA4**			555.33	1.726	1.9	1.300	4.9
**HA5**			562.67	1.714	1.9	1.290	5.1
**HA6**			543.00	1.704	2.0	1.271	5.3

Table S2. Turbidimetric titration for hydrophobized HA in HEPES buffer

Sample	Conc. (%)	Mucin	Turbidity	Absorb. 500 nm	Transmit. 500 nm	Absorb. 660 nm	Transmit. 660 nm
Mucin III	1	---	601.7	1.730	1.8	1.314	4.8
**HA1-C12**	0.15	---	2.06	0.006	98.7	0.003	99.2
	0.3	---	79.97	0.010	97.6	0.007	98.6
	0.5	---	27.10	0.017	95.9	0.011	97.4
**HA2-C12**	0.15	---	13.03	0.005	98.8	0.003	99.2
	0.3	---	57.4	0.013	97.0	0.008	98.1
	0.5	---	91.6	0.024	95.3	0.013	96.9
**HA3-C12**	0.15	---	0.8	0.003	99.3	0.002	99.5
	0.3	---	65.9	0.004	99.1	0.003	99.2
	0.5	---	2.1	0.005	99.1	0.002	99.5
**HA1-C12**	0.15	Type III	581.0	1.682	2.1	1.193	6.2
	0.3		587.3	1.704	2.0	1.220	6.2
	0.5		557.3	1.649	2.3	1.164	6.9
**HA2-C12**	0.15			1.713	2.0	1.227	6.0
	0.3	Type III	574.0	1.688	2.1	1.203	6.3
	0.5		Out of range	**2.043**	0.9	**1.590**	2.7
**HA3-C12**	0.15			**1.718**	1.9	1.238	5.8
	0.3	Type III	Out of range	**1.921**	1.2	**1.470**	3.4
	0.5		Out of range	**2.277**	0.6	**1.868**	1.5

S3. Mucoadhesion studies

Viscosity measurements were carried out in a rheometer Kinexus Pro+ (Netzsch) with a cone-on-plate measuring device was used. Cone had a defined geometry of CP/60 L0881 SS and plate was defined as a disc, where samples were applicated. Flow curves were determined at 25 or 37°C, applying increasing shear rate of 1 to 100 s^{-1}. The viscosity was evaluated for HA, HA-C12 and OTC in trehalose or HEPES buffer and diluted with a mucin dispersion (type II or III) at concentration 5 wt.%. Samples of HA and HA-C12 were dissolved in the respective buffer and stirred for 24 hours on a magnetic stirrer. The solutions of HA, HA-C12 and eye drops were diluted (1:1) with mucin for mucoadhesive samples or with buffer for non-mucoadhesive testing. The samples were incubated at 37 °C for 60 min.

The parameter rheological synergism (ηb) was calculated using apparent viscosity at 100 s^{-1} shear rate [1], according to **Eq. 1:**

$$\eta b = \eta t - \eta(m - p) \qquad (1).$$

where ηt is the viscosity of the mucin-polymer solution system, and ηm and ηp are the viscosity at 100 s^{-1} the mucin dispersion and HA solution, respectively. Measurements were performed at three different concentrations: 0.1, 0.2 and 0.3 wt.%.

The mucoadhesiveness of the HA and HA-C12 was evaluated by rheology. The following samples were prepared for each determination: i) a suspension of mucin (1 wt. %) in HEPES, PBS or trehalose buffers ii) a HA solution in the buffer described in i) at concentration (0.3 wt.%). iii) a suspension mucin (1.0 wt.%) and the tested formulation at concentration (0.3 wt.%). A flow curve in the range of 1–1000 s^{-1} was registered at 25 °C for each sample. The rheological synergism parameter (ηb) was calculated by Eq. (1), from the viscosity coefficient values for each polymer on fixed shear rate (100 s^{-1}) [1]. The samples 1, 2, and 3 were prepared as follows. Sample 1): mucin type III was hydrated with distilled water to a final concentration of 1 wt. % (10 h, 300 rpm, rt). Sample 2): HA-C12 was dissolved at the desired buffer as sample 1. Sample 3): a mucin type III solution (1 wt. %) was suspended in the respective buffer. The HA/mucin mixtures were incubated for 1 hour at 37°C. The measurements were performed in triplicate and reported as a mean value.

Table S3. Rheological characterization of **HA2-C12** in HEPES, trehalose-containing buffer (T) and saline buffers determined at 25°C

Sample	Conc. %	Viscosity (mPa,s) γ = 1/s	Viscosity (mPa,s) γ = 10/s	Viscosity (mPa,s) γ = 100/s	Viscosity (mPa,s) γ = 1000/s
**HA2-C12 HEPES**	0.1	4.5	3.9	3.8	3.6
	0.2	7.7	7.5	7.4	6.5
	0.3	13.8	12.7	12.3	10.1
	0.5	37.1	31.8	28.5	19.5
**HA2-C12 (T buffer)**	0.1	7.2	3.6	3.3	3.2
	0.2	16.8	9.1	7.5	6.2
	0.3	30.4	24.5	18.8	12.0
	0.5	555.7	453.8	103.2	28.2
**HA2-C12 (0.9% NaCl)**	0.1	22.3	16.3	11.7	11.9
	0.2	412.4	540.6	80.0	12.0
	0.3	603.9	540.0	69.3	12.2

0.5	18030.0	2964.0	321.3	42.8

Table S4. Sterilization by filtration using filters of different compositions.

Filter	Pore size of filter	Membrane	Filtration capacity		% recovery after filtration
			HA-2C12	HA	
Sartorius Minisart NML®	0.2 µm	cellulose acetate	Very good	Very good	60-85
Ophtalsart©	0.2 µm	cellulose acetate	Very good	Very good	80-95
Optex®	0.2 µm	Mix of cellulose esters	good	Very good	55-85
PES	0.2 µm	PES Polyethersulfone	difficult	Very good	60-85
Minisart® SRP25	0.2 µm	PTFE Polytetrafluoroethylene	Very difficult	+/-	0

Table S5. Determination of viscosity HA0-C12 after sterilization by filtration using Ophtalsart filter.

Entry	HA2-C12 %	Viscosity (mPa,s) γ = 1/s	Viscosity (mPa,s) γ = 10/s	Viscosity (mPa,s) γ = 100/s	Viscosity (mPa,s) γ = 1000/s
1	0.1	5.4	2.9	2.7	2.6
	0.1-F	6.7	2.9	2.6	2.5
2	0.18	10.2	5.7	5.1	4.3
	0.18-F	9.3	5.4	4.8	4.2
3	0.24	15.4	9.7	8.1	6.4
	0.24-F	11.1	5.9	5.2	4.5

F= means the value of viscosity after filtration.

References

[1] G. Horvát, M. Budai-Szűcs, S. Berkó, P. Szabó-Révész, J. Soós, A. Facskó, M. Maroda, M. Mori, G. Sandri, M.C. Bonferoni, C. Caramella, E. Csányi, Comparative study of nanosized cross-linked sodium-, linear sodium- and zinc-hyaluronate as potential ocular mucoadhesive drug delivery systems, Int. J. Pharm. 494(1) (2015) 321-328.
